# The British Medical Association Meeting at Brighton

**Published:** 1913-08-02

**Authors:** 


### THE BRITISH MEDICAL ASSOCIATION MEETING AT BRIGHTON.

### A Great Address by Sir Berkeley Moynihan.

At previous British Medical Association meetings it has sometimes happened that the addresses in various sections have been more platitudinous than plangent. This year certainly no such mild reproach can be levelled; and a high level of interest and scientific excellence has been maintained throughout the professional sections of the week's work. One address, however, stands out beyond the others, that in surgery, which Sir Berkeley Moynihan delivered, entitled "The Gifts of Medicine to Surgery." The eminence of this Leeds surgeon has long been unquestioned and unquestionable; and his prolific writings have also been notable for clear thinking and correct speaking. But this particular address must raise its author, we do not hesitate to say, still higher in the estimation of his colleagues, for it is a work of genius.

### Sir Berkeley's Record.

Professor Sir Berkeley Moynihan, as is well known, is one of the leaders of the Leeds School of Surgery, where the influence and example of Mayo Robson, we may assume, assisted in directing his bent towards abdominal surgery, and especially towards the surgery of the upper abdomen. Notwithstanding the claims of a large practice as a consulting surgeon, as a hospital surgeon, and as a teacher, he has done much valuable research into the problems of gastric ulcer, gallstones, and similar diseases, which has brought him world-wide recognition as an original worker, a patient investigator, and a pioneer surgeon. With the possible exception of Sir Arbuthnot Lane, there is no British surgeon whose reputation stands higher in surgical circles on the Continent and across the Atlantic. Moreover, he possesses the rare gifts of lucid exposition and an excellent English style, and has put them to good use in innumerable contributions to medical literature.

It is not surprising, therefore, that Sir Berkeley Moynihan has during the last ten years or so come gradually to be regarded by the rank and file of the profession as a teacher to be trusted. During that time he has, in Kipling's words, "kept his light so shining a little in front of the rest" in connection with the surgery of the upper abdomen; and no small share in the very great advances of this branch of surgery is due to him. The address at the British Medical Association meeting not only sums up very admirably the present position of surgical (and medical) science as it applies to the diseases of these regions: it does more than that, for it traces the causes of past progress and the probable lines of future advance.

### Anoci-Association.

Among the most important of the many absorbing topics broached is that of the prevention of shock, and of Dr. G. W. Crile's "Anoci-Association" hypotheses concerning it. Crile's views have been quite recently fully stated to the medical profession; and will be further discussed at the forthcoming International Medical Congress. They are, in addition, complex and highly technical; and we do not for all these reasons propose to discuss them here. The point we wish to bring out is that Sir Berkeley Moynihan believes that these hypotheses contain such a substantial framework of truth that they are safe to work on; and he himself has been putting them into practice for some months with results which he deems satisfactory.

This position is the more remarkable in that Crile's theories are still strongly combated by other investigators in the United States—the country of their origin. The hypotheses of Yandell Henderson, another American, are the direct negation of those of Crile; and as a matter of fact, opinion in Great Britain has been steadily moving against Crile in this matter for the last two or three years, and in favour of Henderson. British surgeons seem inclined to treat the latest developments of Crile's work, the "anoci-association" hypotheses and the technique based upon it, as too fanciful in theory and too complicated in practice. Decidedly the last words on this question will not be spoken for some years yet; and in spite of his ingenuity in experiment, Crile's brilliant work has not always turned out absolutely solid.

This attitude of scepticism is eminently rational provided it is accompanied by a willingness to put the matter to practical tests. Crile's system of preventing shock may be laborious and complicated; that is a reason for receiving it with caution, but is no excuse for refusing to give it a trial. Sir Berkeley Moynihan believes in it, rightly or wrongly; his past performances and his penetrating insight into the problems of surgery are such that his advocacy is bound to receive attention. To speak the truth, we shall not be surprised to find him in a year or two abandoning part of his present position; but, on the other hand, he may be impregnably fortified in it. What the surgical profession has to do is to put the matter to the touch without delay; and even if the verdict ultimately be unfavourable to Dr. Crile and Sir B. G. A. Moynihan, they will still have the credit of having sought after truth diligently to the best of their abilities.

### The Debt of Medicine to Surgery.

This exposition of Crile's work occupies, however, only a small part of the address, the major portion of which was devoted to the development of the theme suggested by the title. As Sir Berkeley unfolded the steps of advance in our knowledge of gastric ulcer, appendix dyspepsia, cholelithiasis, and many other conditions about which current teaching has undergone such radical changes in the last decade and a half, even the most old-fashioned physician must have felt compelled to acknowledge the reality of the debt which medicine owes to surgery, and the world at large to both. What Sir B. Moynihan calls the "pathology of the living" has indeed thrown shafts of light upon the obscurities of many abdominal diseases; and another of his striking phrases is that in which he applauds the "surgeon who walks by sight, not by faith."

Throughout his address this distinguished surgeon avoids that egotistic vein which seems to be the besetting sin of a good many of the lesser men in this specialty. To this tendency Sir Berkeley Moynihan is easily superior; and his frequent mention of the original work of other surgeons contrasts with his modesty in suppressing references to his own share in the researches which he summarises. His address is profound in conception, masterly in execution, and a model of what a scientific essay should be in composition.

The Address in Medicine.

The companion address in the other fundamental division of the healing science, medicine, was delivered by Professor G. R. Murray; and this, too, was a thoroughly sound and scholarly piece of work. In reviewing the present state of our information about the ductless glands and of current views as to the internal secretions, Professor Murray did well to emphasise the distinction between exact knowledge and rash speculation. This warning is the more necessary because some of the developments of organo-therapy—the thyroid treatment of cretinous idiots, for instance—have been so spectacular that a great temptation is offered to inexact generalisations. While quite properly cautious in his teachings, and more concerned at present to lay the foundations truly than to rear an imposing superstructure on them, Professor Murray obviously looks forward to a not distant date when the complete understanding of the ductless glands and of internal secretions in general will add weapons of great precision and potency to the armamentarium of the physician.

Other Notable Papers.

The super-excellence of these addresses is no reflection upon the quality of a great many other papers; for there were many valuable discussions. In the section of medical sociology and State medicine this was especially true; Professor Moore's paper and the debate following it are dealt with elsewhere in this issue (p. 517). To this section Mr. Bernard Shaw discoursed in his usual vein of bantering omniscience, and told a story about an alleged uncle of his, a member of the medical profession, who, when asked what was the lowest fee he would accept, replied that the Mint had not yet coined anything small enough for him to refuse. This grim repartee obviously amused Mr. Shaw's audience, many of whom seemed able to appreciate the fidelity to life of the description and the circumstances.

The Section of Neurology found itself entangled in theological matters, as the outcome of a paper by Dr. T. A. Williams on "Truths and Fallacies of Mental Healing," in which the question of the value of the Confessional from a psycho-therapeutic standpoint was raised. Dr. Long committed himself to the statement that among Roman Catholics the incidence of insanity is only about 50 per cent. of what it is among Protestants, and ascribed this to the attention paid to the psychical side of their diseases. Confession, in short, according to Dr. Long, is not only good for the soul but for the body too. A subsequent speaker, who had had asylum experience in Ireland, denied the truth of Dr. Long's facts about the relative proportions of insane Catholics and Protestants. The sense of the meeting was well summed up by another doctor who described how a shrewd old country practitioner used to invite selected patients to come to his consulting room for the luxury of a good cry.

Some very unusual, not to say diverting, contributions were made to the debate on Crime and Punishment. Dr. Mercier and Dr. Scott (late Governor of Holloway Prison) were in favour of punishment as repressive of and deterrent to crime. The latter holds that there is too much mawkish sentimentality nowadays in dealing with criminals; and related many interesting examples of the way in which the spread of education is leading to cleverer crimes, in which all sorts of scientific inventions are made use of. Prince Peter Kropotkin, who said he was "an old gaol bird with ample experience of prison life in Russia and France," declared that imprisonment has not the deterrent effect that is claimed for it. A Suffragette ex-prisoner, Miss Allen, also struck a personal note by describing an unaccountable desire for alcohol while in Holloway Prison, to be gratified by drinking green Chartreuse on her release. We may take leave to doubt whether either of these "gaol birds" is typical of the ordinary criminal, who probably responds quite differently to deterrent punishments.

An Attack on "Punch."

The National Temperance League has been very much in evidence throughout the meeting; and a great many doctors have addressed lay gatherings in various places of worship in and near Brighton on the evil effects of alcohol. Mrs. Scharlieb in particular distinguished herself by a senseless attack on *Punch*. She holds that the typical figure of John Bull, as depicted in *Punch* cartoons, is that of a man who is by no means a total abstainer. In this respect she is no doubt right; but she goes on to complain that such pictures deprave the rising generation and are a "distinct incitement to evil." With all respect to Mrs. Scharlieb, this is nonsense: the incitement to evil in *Punch's* conception of John Bull exists in her mind, not in the minds of children or in actual fact. Even if the John Bull cartoons did deprave anyone, *Punch* could at least reply that he draws that individual from life, not from Mrs. Scharlieb's ideal of what he ought to be. Save for this false note, the Brighton meeting of 1913 must be held to have been one of the most successful, from the scientific and technical point of view, of any in the series.

